# A qualitative study of health information technology in the Canadian public health system

**DOI:** 10.1186/1471-2458-13-509

**Published:** 2013-05-25

**Authors:** Kate Zinszer, Robyn Tamblyn, David W Bates, David L Buckeridge

**Affiliations:** 1 Clinical and Health Informatics Research Group, McGill University, 1140 Pine Avenue West, Montreal, QC H3A 1A3, Canada; 2 Harvard Medical School, 25 Shattuck Street, Boston, MA, 02115, USA; 3 Agence de la Santé et des services Sociaux de Montréal, 3725 Saint-Denis Rue, Montréal, QC H2L 1M3, Canada

**Keywords:** Health information technology, Electronic infrastructure, Informatics, Surveillance, Public health, Canada

## Abstract

**Background:**

Although the adoption of health information technology (HIT) has advanced in Canada over the past decade, considerable challenges remain in supporting the development, broad adoption, and effective use of HIT in the public health system. Policy makers and practitioners have long recognized that improvements in HIT infrastructure are necessary to support effective and efficient public health practice. The objective of this study was to identify aspects of health information technology (HIT) policy related to public health in Canada that have succeeded, to identify remaining challenges, and to suggest future directions to improve the adoption and use of HIT in the public health system.

**Methods:**

A qualitative case study was performed with 24 key stakeholders representing national and provincial organizations responsible for establishing policy and strategic direction for health information technology.

**Results:**

Identified benefits of HIT in public health included improved communication among jurisdictions, increased awareness of the need for interoperable systems, and improvement in data standardization. Identified barriers included a lack of national vision and leadership, insufficient investment, and poor conceptualization of the priority areas for implementing HIT in public health.

**Conclusions:**

The application of HIT in public health should focus on automating core processes and identifying innovative applications of HIT to advance public health outcomes. The Public Health Agency of Canada should develop the expertise to lead public health HIT policy and should establish a mechanism for coordinating public health stakeholder input on HIT policy.

## Background

Health information technologies (HIT) provide the potential for greatly enhanced public health surveillance and response capacities, but the fragmented nature of health data limits the potential for HIT to enhance public health practice. This fragmentation is a consequence of the inconsistent use of standards for data storage and transmission [1] as well as perceived and real legal and privacy barriers to data sharing. The need to integrate large volumes of health information in a timely manner to guide practice is a key motivation towards improving the use of HIT in public health [2]. Public health HIT sophistication and adoption have advanced rapidly over the past decade in certain countries including Sweden [3], the Netherlands [4] and Germany [5]. Enhanced public health HIT infrastructure is critical for successful adoption of the 2005 International Health Regulations (IHR 2005), which emphasize improved surveillance infrastructure [6]. However, the development and broad adoption of HIT in public health settings worldwide, including Canada [2], still face considerable challenges [7,8].

The need for improved public health surveillance infrastructure in Canada has been recognized for some time. In 1997, a report from the National Forum on Health recommended the creation of a nationwide population health information system [9], a recommendation the Advisory Council on Health Infostructure also endorsed [10]. In 2001, Canada Health Infoway (CHI) was established as a non-profit, independent shared governance corporation funded by the Government of Canada [11]. The initial focus of CHI was to accelerate the implementation of the electronic health record (EHR) across Canada and to produce an EHR blue print [12]. CHI was founded with a $500 million federal investment and it has received another $1.5 billion since that initial investment [13]. The 2003 SARS crisis renewed concerns about the gaps in HIT use in public health and the Naylor report recommended the development of a national public health surveillance system [14]. This recommendation was added to the mandate of CHI in 2004 [8] and provided the impetus for creating the pan-Canadian Public Health Surveillance System (Panorama) along with $150 million to develop an information system that would support public health professionals across the country to manage cases of reportable communicable diseases and the delivery and tracking of vaccines [15].

Despite a substantial federal investment in HIT, Canada continues to lag behind other countries in the adoption of public health electronic health information systems [3-5,16]. The objective of the current study was to describe the influence of HIT policy on the Canadian public health system and to identify successes and challenges, and to suggest future directions to improve the adoption and effective use of HIT to advance public health in Canada.

## Methods

This study was part of a larger research initiative, which assessed the effectiveness of the Canada e-health plan and identified ways to increase electronic health record adoption in Canada [17,18]. A qualitative case study design was employed using interviews with informants who were responsible for leadership, policy, or information technology in their organization. Selected organizations were from one of four stakeholder groups influential in the adoption and use of HIT: 1) national and provincial agencies responsible for health information technology, 2) quality and safety or public health agencies, 3) health professional associations, and 4) health information technology vendors. We selected informants from provinces with differing degrees of HIT implementation. Alberta was one of most advanced provinces in terms of EHR adoption, while British Columbia was average, and Ontario lagged behind other provinces [19].

Based upon prior research [20-24], a semi-structured questionnaire was developed to solicit perceptions of the importance of health information technology (HIT) in public health, to identify challenges in the implementation and use of HIT in public health, and to suggest future directions (Additional file 1). For this study, the term “health information technology” was used to represent both electronic medical records and electronic health records. An electronic medical record is health-related information on an individual from a single organization whereas an electronic health record is the collation of health-related information on an individual across various health care organizations [25].

Those consenting to participate were sent the interview guide prior to the scheduled telephone interview. Interviews were conducted between September and November 2009. The interviews typically lasted 45 minutes and were taped with the consent of the informants.

The interviews were transcribed and the text was analysed using grounded theory principles of coding and theme abstraction [26], although we did identify two categories (importance of HIT policy for public health and current HIT impact in public health) *a priori*. From the interview transcripts, a series of codes were developed and then grouped into similar concepts. These concepts were then combined to form categories (challenges, future directions) or were assigned to categories identified earlier in the study (importance of HIT policy for public health and current HIT impact in public health, challenges, future directions). The initial coding framework was based upon the interview guide, and as the coding proceeded, additional concepts and themes emerged. The concepts defined the emerging themes within or between each category, and formed the basis of a framework for the perspectives of HIT and HIT policy in public health. An example of the coding framework is provided in Additional file 1. The codes, categories, and themes were derived through iterative readings and discussion of the transcripts by three authors (KZ, RT, and DB). Quotes from the interview transcripts that might identify informants were masked to protect confidentiality. The McGill University Institutional Review Board approved the protocol for this study.

## Results

Of the 32 informants asked to participate, 8 declined to answer questions about public health as they felt they had insufficient expertise to comment on the adoption and use of HIT in public health. Among the 24 informants who responded to the public health section of the interview, 8 represented safety and quality or public health agencies, 7 represented agencies responsible for health information technology, 5 represented health professional groups, and 4 were from the vendor community. Eleven of the informants were from national organizations with 6 from Ontario, 5 from Alberta, and 2 from British Columbia.

### Importance of HIT policy for public health

Despite their different disciplines, the informants were largely in agreement that HIT policy was highly significant for public health practice and that HIT policy should improve public health outcomes.

It was felt that HIT is crucial for public health practice, which relies on data and information derived from clinical settings to support decision making. SARS was given as an example by informants of how a lack of HIT policy can have serious implications for public health.

“We’ve learned the hard way in Canada, that when you don’t have integration of those records, it’s very, very difficult and you make mistakes, right? And public…there’s nothing worse than a public health crisis.” HIT agency, Respondent #21

A key benefit of HIT policy is that it allows the integration of information, record linkage, from different agencies and partners, and improves the timeliness of communication and coordination of a public health response.

“This is an old cliché…but if you can’t measure it, you can’t manage it…HIT has the ability to allow us to monitor, to track, to measure and to report.” Health professional, Respondent #27

One informant disagreed that HIT policy was important for public health practice, stating that HIT policy in public health does not deserve attention at the moment as there are more relevant issues in HIT policy, such as clinical HIT policy.

“Last thing we need is further scope and spread on the HIT policy, when we are still dealing with foundation and building block components. This, ultimately, should become the ultimate prize of aggregated, critical, mass of information related to jurisdictions/populations that can be mined and alerted.” HIT agency, Respondent #24

### Current HIT impact in public health

The current state of HIT in public health was also discussed. Opinions were variable, ranging from no impact and poorly done to having an enormous impact on the management of public health issues. Positive perspectives attributed to HIT policy were increased standardization of data and processes and an improved awareness of the need for system interoperability. Additionally, the benefits of increased communication among various organizations and the ability of public health professionals to access lab results were other positive impacts of HIT policy.

The perceived negative aspects of HIT policy in public health included a lack of results from current investments and a tendency to let technology, as opposed to practice needs, define policy. It was also stated by some informants that the public health applications of HIT were not recognized from the beginning by Canada Health Infoway and this lack of consideration has hampered the identification and development of linkages or integration between EHR and public health information systems.

“That [Panorama] is one of the few, where the jurisdictions have agreed that we have to be national rather than jurisdictional. The concept is very good but it’s not yet at the point of its expected delivery. But we have the right idea in the making, understanding that public health has no border.” Health professional, Respondent #15

“It’s an interesting phenomena, it [public health] really does seem to have a separate existence, outside of discussions in the health system. It comes as an afterthought, or a separate thought. No matter how many times we have examples of public health affecting the health and safety of Canadians.” Quality/safety/public health agency, Respondent #20

“That was largely the phenomena of the vendor bidding [Panorama], on something they couldn’t deliver, and we had a number of problems coming from that. And, and I guess that’s the other thing that Infoway has been able to do, which is to become a sort of source of knowledge about what’s realistic, in terms of the vendor community… I’ve used the phrase “bait and switch” in a room full of vendors and no one gets upset, which gives you some idea of kind of their view of the world, which is true. We’ll say we can build it, and we’ll try and build it, and we might or not… might or might not be able to build it particularly on time or on budget. So, it’s a bit of a voyage of exploration.” Quality/safety/public health agency, Respondent #17

Some respondents stated that Panorama is a response to the past, and has not considered the new opportunities for more rapid public health surveillance that are enabled through the implementation of EHRs. Respondents noted that surveillance practices have not evolved and that investments in systems, like Panorama, are automating inefficient practices as opposed to using IT to improve practice. Another issue raised was the perception that HIT policy for public health has received too much focus at the federal level and insufficient focus at the provincial and local levels.

“…and even now, we have the separation [of public health and EHR]. So, Panorama’s got an immunization module within it, it’s actually set out to replicate what public health does now, in many jurisdictions, where family physicians and paediatricians do the immunizations of babies and then, public health catches up with the information when children enter school…Now, with an electronic health record, you could just get that information as the needles go into the babies arms and feed it to public health. So why have a whole separate system? We haven’t tried to reconceptualise the way public health does that.” Quality/safety/public health agency, Respondent #29

### Challenges

Several challenges in HIT policy implementation were also mentioned by the informants. A consistent challenge that emerged was the complexity of implementing a national HIT public health policy given the differences across jurisdictions in public health infrastructure. Insufficient funding in public health HIT as well as the underfunding and neglect of public health in general were also noted as important challenges to HIT policy. The reliance in public health on unproven systems and paper-based processes were also perceived barriers to HIT implementation. It was also mentioned that locations in Canada with the most pressing public health problems tend to be the jurisdictions with the worst technology and that HIT is doing little to help improve the health in these areas.

“I’m not sure that they [Public Health Agency of Canada] have a strong plan right now, and I think it has more to attribute to the newness of the agency, and the fact that they have not, in my view, put out a comprehensive plan with a clear direction of actions that are required to achieve what they’re trying to do. I think getting the agency to set out a clear line of action, getting the provinces to support it. And again, it’s gonna take funding from probably the national level, to make this work really well, because the provinces won’t give it a priority, except at their local levels, unless the national health agency does something.” Quality/safety/public health agency, Respondent #22

“Public health agencies are still relying on their conventional paper manual processes, to manage most things… there’s some early implementation and adoption of technology, but it’s not nearly where it should be.” HIT vendor, Respondent #6

“I think yes you need strong public health, yes it needs health information to back it up. But the more I see of Panorama, I sort of think it’s just an exercise in stuffing a lot of old data into a database for what purpose? The places with the worst public health issues in Canada are the places with the least amount of health information technology. So, remote communities, first nation communities…its [computerizing] not gonna make the water any cleaner.” Quality/safety/public health agency, Respondent #17

### Future directions

Numerous suggestions were made about potential directions for HIT in public health. It was recommended that a comprehensive strategy be established with clear direction and commitment of funding, something that requires national policies and leaders. The need to work on information sharing between jurisdictions while respecting existing legislation and policy was also mentioned by informants. Informants voiced concerns that there should be a public health component to all HIT, including advancements in interoperability of clinical and public health systems as well as consumer engagement indisease surveillance and in reporting.

“A model for public health surveillance and public health engagement that could involve some central control body…cascading system down to provinces and to regions, and more locally.” Quality/safety/public health agency, Respondent # 11

“I would like to see much more serious engagement between the Public Health Agency of Canada and CHI…The Public Health Agency of Canada and Infoway need to be discussing their common platforms, their common data sources… their data sharing practices, etc., MUCH more than they currently do” Quality/safety/public health agency, Respondent # 26

“And so, it’s a system that’s… I don’t know how many years behind? So it’s being built to respond to a way public health worked, back in Trudeau. And so, neither takes account of the evolution of public health services in this country, or the evolution of technology. I’d actually like to see systems being built in a way that integrates what’s happening on the clinical/institutional side with public health. And so with something like communicable disease control, investigation of outbreaks, to bring the data from all these different providers together with what public health holds. And to be able to help manage the communications in an outbreak, which would really mean integration across healthcare settings and providers. So, I would really say you’ve gotta be prepared to throw away some of the work that’s been done, and start over.” Quality/safety/public health agency, Respondent # 29

It was mentioned that public health focuses on improving the health of populations, which requires primary care data but also data collected outside of the realm of medical practice in fields such as nutrition, climate, and environmental health. With more comprehensive data, public health could track the performance of public health programs to support planning, implementation, and monitoring of strategies.

## Discussion

There was nearly unanimous agreement among respondents that public health must be considered explicitly when developing HIT policy. Current benefits of HIT identified by the respondents included progress in the standardization of data and processes, advancements in the interoperability of systems, and associated improvements in communication among jurisdictions. Existing barriers and challenges to the effective use of HIT in public health included the lack of a shared national vision and leadership, uncoordinated policy, insufficient investment, and poor conceptualization of the role of HIT in public health. Future directions suggested by respondents included the establishment of national leadership in public health IT, a renewed focus on the interoperability of clinical and public health systems within regions and provinces, and the development of innovative strategies for use of EHR data to further public health objectives.

### HIT system design

Data sharing between clinical and public health settings is critical for effective public health practice. Currently, data sharing tends to be slow and incomplete due to a reliance on manual approaches [27]. The adoption of a single public health system such as Panorama by all public health jurisdictions is neither necessary nor sufficient to ensure timely and accurate data sharing through HIT. Moreover, a top-down approach to HIT at a national scale has failed in some countries, notably the UK, due to complexity, inflexibility, and an inability to meet local needs [28,29]. HIT will facilitate data sharing if public health systems and clinical systems are interoperable in the sense that clinical systems are able to record and transmit in a standard manner to public health systems the data required by public health agencies. In the Canadian healthcare system, there are currently few incentives for software vendors to build, and for clinicians and healthcare institutions to purchase, clinical systems that are interoperable with the systems in public health agencies. Concerted federal and provincial policies to encourage or require the adoption of clinical systems that can capture and transmit specified data elements to public health in a standard manner would help to advance data sharing and innovation in public health practice.

### Surveillance

Public health agencies may be able to realize an immediate benefit from automating current manual processes, although such automation will usually entail careful study and modification of existing processes. Two such applications highlighted by the ‘Meaningful Use’ (MU) policy initiative in the United States are reportable disease surveillance and immunization registries [30]. The MU initiative provides incentives to eligible healthcare providers and institutions who purchase HIT systems certified to support precisely defined functions, and they include specific requirements for transmission of laboratory and immunization data from clinical settings to public health agencies. Even greater potential benefit, however, may be realized if current public health practices are reassessed in light of public health objectives and the opportunity presented by the adoption of HIT in public health and clinical settings. One such example is the near real-time monitoring of health care utilization patterns by public health agencies, or syndromic surveillance, which can play an important role in the rapid detection and characterization of public health emergencies of domestic and international concern [31]. Novel approaches, however, must also be conceptualized, piloted, and evaluated. A broader culture of innovation in public health and sustainable funding for applied public health informatics research is critical to support the effective use of HIT to modernize public health practice and advance public health objectives. The Beacon community funding through the US Office of the National Coordinator (ONC) for HIT is an example of one programmatic approach to supporting such applied research [32]. Support for similar ‘bottom-up’ initiatives should identify keys in a Canadian context to effective local and regional data sharing between clinical and public health settings.

### Governance

Leadership and coordination regarding HIT in public health were identified as being important and requiring improvement. The public health community in most countries is relatively small in comparison to the clinical care community, which drives the vast majority of the investment in HIT. In this context, the HIT needs of public health are more likely to be met if they are supported broadly within the public health community and articulated clearly by effective leaders. The Centers for Disease Control and Prevention in the United States has created the Public Health Surveillance and Informatics Program Office (PHSIPO) to provide leadership and coordination in the development and use of HIT in public health practice [33]. PHSIPO also supports the Joint Public Health Informatics Task Force [34], which coordinates input on HIT from public health stakeholders, and PHSIPO works closely with the ONC to ensure that public health needs are considered in the creation and implementation of all HIT policies. An exact replication of such entities is not necessarily the optimal approach in all countries, but in Canada there should be improved support for public health stakeholder policy input, a federal focus of expertise within the Public Health Agency of Canada, and close communication between these public health entities and provincial and federal HIT policy makers and funders. Improved leadership and coordination should help to accelerate the adoption and effective use of HIT in public health and to align clinical and public health needs in the development of HIT policy more broadly.

### Study limitations

The results represent neither the opinions of all stakeholders nor all perspectives on public health HIT in Canada. Our target participants were senior professionals or administrators, excluding end-users of HIT in public health, such as nurses, doctors, public health inspectors, epidemiologists, analysts, and clerical staff. These views would provide valuable insight into HIT in public health and associated recommendations, which should be explored in future research.

## Conclusions

HIT has the potential to play a key catalytic role in advancing public health objectives in Canada. To realize that potential, we recommend the development of policies to drive the adoption of clinical systems that share defined data with public health agencies. The application of HIT in public health in the short-term should focus on automating core processes and in the longer-term sustainable funding should support innovation and evaluation to identify novel applications of HIT to advance public health outcomes. Finally, we propose the establishment of a clear focus of public health HIT leadership within the Public Health Agency of Canada and a mechanism for public health stakeholder input on HIT policy to support the alignment of clinical and public health HIT needs and the effective adoption and use of HIT in public health practice.

Given the global importance of public health HIT development and adoption and the shared challenges of strengthening links between clinical and public health practice, we believe that the Canadian experience is a valuable case study for public health HIT direction and strategy in other countries. The IHR 2005 represents a seminal change in policy for global disease surveillance and control, but without substantial advances in national public health HIT infrastructures, implementation of this policy will be difficult [35]. Each nation faces unique challenges that require tailored solutions for the continued improvement of HIT in their public health organizations, but many of the issues and potential solutions identified in a Canadian context should be relevant to other countries.

## Abbreviations

CHI: Canada Health Infoway; EHR: Electronic health record; HIT: Health information technology; MU: Meaningful use; ONC: Office of the National Coordinator; IHR 2005: 2005 International health regulations; PHSIPO: Public Health Surveillance and Informatics Program Office.

## Competing interests

The authors declare that they have no competing interests.

## Authors’ contributions

All authors contributed to the study concept and design, and critically revised the manuscript for important intellectual content and approved final version submitted for publication. KZ, RT and DB contributed to the analysis, interpretation of the data, and drafted the manuscript.

## Pre-publication history

The pre-publication history for this paper can be accessed here:

http://www.biomedcentral.com/1471-2458/13/509/prepub

## Supplementary Material

Additional file 1 Coding example illustrating codes, concepts and categories that emerged from the data. Click here for file
