# Supplementary material for: A qualitative study of health information technology in the Canadian public health system
Source: BMC Public Health. 2013 May 25;13:509. doi: 10.1186/1471-2458-13-509 (PMC3665446; doi:10.1186/1471-2458-13-509)
Supplement: Additional file 1 — Coding example illustrating codes, concepts and categories that emerged from the data. [file 1471-2458-13-509-S1.doc]

Additional file 1

1) **In your opinion, what is the significance of HIT policy on Public Health practice and outcomes? Can you please elaborate?**

2) **In general, would you say that the Canadian HIT policy appears effective with respect to the promotion of public health practices and outcomes?**

3) **Are you aware of HIT initiatives related to public health that are in place today in your jurisdiction? If yes, which ones?**

| Data | Code | Concept | Category |
| --- | --- | --- | --- |
| "On things like food-borne outbreaks or clusters of infectious disease, by and large, I think we’re still in the Stone Age where the public health officials rely on reports from labs for sentinel physicians, that come by fax or maybe email". | Perceived outdated practices | Require modernization of practices | Potential of HIT in public health |
| "I think that the significance (of HIT policy on public health practice and outcomes) is very high, but I'm not sure that there's a reality of policy around HIT with public health practice…I think that it's a neglected part of the health system when we talk about EHRs" | Perceived neglect of public health in EHR discussions  Perceived importance of HIT policy in public health | Require involvement of public health in EHR discussions/planning  HIT policy is necessary in public health practice | Challenges of HIT in public health  Importance of HIT policy for public health |
| “What would I change [HIT policy]? I would have a public health component to the Canadian HIT policy that would include that in the interoperability so we do have an accurate record of all public health parameters incorporated into the EHR" | Requesting interoperability between health care systems | Integration of different data sources for the EHR | Potential of HIT in public health |
| "I’d prefer to, I’d actually like to see systems [HIT systems] being built in a way, that integrates what’s happening on the clinical/institutional side with public health. And so, with something like communicable disease control, investigation of outbreaks, to bring the data from all these different providers together with what public health holds. And, and to be able to help manage the communications in an outbreak, which would really mean integration across healthcare settings and providers. So, I think... I would really say you’ve gotta be prepared to throw away some of the work that’s been done, and start over.” | Requesting the integration of different health care providers and public health in current HIT systems | Integration and application of different data sources in public health practice | Potential of HIT in public health |
